# Supplementary material for: Awareness and knowledge of Chikungunya infection following its outbreak in Pakistan among health care students and professionals: a nationwide survey
Source: PeerJ. 2018 Aug 30;6:e5481. doi: 10.7717/peerj.5481 (PMC6119596; doi:10.7717/peerj.5481)
Supplement: Supplemental Information 1 [file peerj-06-5481-s001.docx]

**Knowledge and Awareness of Chikungunya Infection among Health Care Professionals in Pakistan**

**Gender:** □ Male □ Female

**Age**: _______________

**Working Status**: □Student □Working □Unemployed

**Level of Education**: □Graduation □ Post graduation

**Field of Education**: □Pharm-D □MBBS □BDS □Physiotherapy □Nursing

**Province**: □Punjab □Sindh □Baluchistan □KPK □FATA □JB □AJK

**General Awareness**

1. **Have you ever heard about Chikungunya Infection?**
2. Yes 1
3. No 0
4. **From where you heard about chikungunya?**
5. Television 0
6. Radio 1
7. Newspaper 2
8. Social Network/Media (Facebook, Twitter, Whatsapp) 3
9. Health Seminar/Program/Workshop 4
10. Teachers 5
11. Family 6
12. Friends 7

**Knowledge of Recent Outbreak in Pakistan**

1. **Do you know chikungunya outbreak has been reported in Pakistan?**
2. Yes 1
3. No 0
4. **If above answer is “Yes” then where chikungunya outbreak has been recently occurred?**
5. Lahore 0
6. Karachi 1
7. Multan 2
8. Faisalabad 3
9. Islamabad 4
10. Don’t know 5

**Basic Disease Knowledge**

1. **Chikungunya is**
2. Bacterial Infection 0
3. Viral Infection 1
4. Don’t know 2
5. **Which infection is closely related to chikungunya infection?**
6. Pneumonia 0
7. Dengue Infection 1
8. Ebola Infection 2
9. Don’t know 3

**Knowledge of vector, spread and transmission of disease**

1. **Chikungunya is caused by mosquito bite, What is the name of mosquito spreading chikungunya?**
2. Anopheles 0
3. Adese 1
4. Both 2
5. Don’t know 3
6. **What is common breeding site of chikungunya mosquito?**
7. Water storage containers/Stagnant water 0
8. Dirty water 1
9. Garbage and Mud 2
10. Don’t know 3
11. **During which time chikungunya mosquitos bite preferably?**
12. Day 0
13. Night 1
14. Anytime 2
15. Don’t Know 3
16. **During which season chikungunya infection is most common?**
17. Dry summer 0
18. Monsoon 1
19. Winter 2
20. Spring 3
21. Don’t Know 4
22. **Does chikungunya infection transfer from human to human contact?**
23. Yes 1
24. No 0
25. Don’t know 2
26. **Does chikungunya infection transfer from mother to new born child?**
27. Yes 1
28. No 0
29. Don’t know 2

**Symptomology**

1. **What are symptoms of chikungunya infection?**
2. Fever Yes1/No0/Not sure2
3. Joint Pain Yes1/No0/Not sure2
4. Muscle pain Yes1/No0/Not sure2
5. Headache Yes1/No0/Not sure2
6. Nausea Yes1/No0/Not sure2
7. Fatigue Yes1/No0/Not sure2
8. Rash Yes1/No0/Not sure2
9. **After the bite of infected mosquito, how many days it took to appear symptoms?**
10. Abruptly (at the same time when mosquito bites) 0
11. 3-7 days 1
12. on next day of mosquito bite 2
13. I am not sure 3
14. **For how many days symptoms of chikungunya last?**
15. One month 0
16. 7-10 days 1
17. One day 2
18. I am not sure 3

**Prevention and Treatment**

1. **Is Chikungunya preventable disease?**
2. Yes 0
3. No 1
4. Not Sure
5. **are there any specific drug available for chikungunya treatment?**
6. Yes 0
7. Not 1
8. Not sure 2
9. **Is there any vaccine available for chikungunya prevention?**
10. Yes 1
11. No 0
12. Not Sure 2
